# The proneurotrophin receptor sortilin is required for *Mycobacterium tuberculosis* control by macrophages

**DOI:** 10.1038/srep29332

**Published:** 2016-07-08

**Authors:** Cristina L. Vázquez, Angela Rodgers, Susanne Herbst, Stephen Coade, Achim Gronow, Carlos A. Guzman, Mark S. Wilson, Makoto Kanzaki, Anders Nykjaer, Maximiliano G. Gutierrez

**Affiliations:** 1 Research Group Phagosome Biology, Helmholtz Centre for Infection Research, Inhoffenstrasse 7, 38124 Braunschweig, Germany; 2 Host-pathogen interactions in tuberculosis laboratory, The Francis Crick Institute, Mill Hill Laboratory, The Ridgeway, London, NW7 1AA, UK; 3 Department of Vaccinology and Applied Microbiology, Helmholtz Centre for Infection Research, Inhoffenstrasse 7, 38124 Braunschweig, Germany; 4 Allergy and Anti-Helminth Immunity Laboratory, The Francis Crick Institute, Mill Hill Laboratory, The Ridgeway, London, NW7 1AA, UK; 5 Graduate School of Biomedical Engineering, Tohoku University, Sendai, Miyagi, Japan; 6 The Lundbeck Foundation Research Center MIND, Department of Medical Biochemistry, Aarhus University, DK-8000 Aarhus, Denmark

## Abstract

Sorting of luminal and membrane proteins into phagosomes is critical for the immune function of this organelle. However, little is known about the mechanisms that contribute to the spatiotemporal regulation of this process. Here, we investigated the role of the proneurotrophin receptor sortilin during phagosome maturation and mycobacterial killing. We show that this receptor is acquired by mycobacteria-containing phagosomes via interactions with the adaptor proteins AP-1 and GGAs. Interestingly, the phagosomal association of sortilin is critical for the delivery of acid sphingomyelinase (ASMase) and required for efficient phagosome maturation. Macrophages from *Sort1*^−/−^ mice are less efficient in restricting the growth of *Mycobacterium bovis* BCG and *M. tuberculosis*. *In vivo*, *Sort1*^−/−^ mice showed a substantial increase in cellular infiltration of neutrophils in their lungs and higher bacterial burden after infection with *M. tuberculosis*. Altogether, sortilin defines a pathway required for optimal intracellular mycobacteria control and lung inflammation *in vivo*.

The process of phagosome maturation is central for the development of the innate and adaptive immune response^1^,^2^. In this process, different subsets of endosomal and lysosomal components are delivered sequentially into the phagosome in a very organized manner. However, little is known about the mechanisms that contribute to the spatiotemporal sorting of luminal and membrane proteins into phagosomes^3^. Many intracellular pathogens have developed mechanisms to hijack the normal trafficking of phagosomes and survive within host cells^4^,^5^. A subpopulation of *Mycobacterium tuberculosis* that it is phagocytosed by macrophages arrests phagosome maturation impairing bacterial targeting to phagolysosomes, a process that contributes to disease^6^,^7^,^8^. However, a clear mechanistic understanding of the dynamics of intracellular *M. tuberculosis* infection and how host defenses are organized against this pathogen is missing.

One process required for the host defense in phagocytes involves the intracellular trafficking of hydrolases to the phagosome from several organelles. In this regard, sortilin, also known as neurotensin receptor 3 (NTR3), is a transmembrane receptor that transports lysosomal proteins from the *trans*-Golgi network (TGN) into lysosomes, as an alternative route to mannose-6-phosphate receptors (MPRs)^9^,^10^,^11^. Sortilin consists of a large luminal domain, a single transmembrane segment and a short C-terminal cytosolic tail. The luminal region possesses a single Vps10 domain^12^ and the cytosolic tail of 53 amino acid residues contains motifs involved in the anterograde and retrograde transport between the Golgi complex and endosomes, similar to the corresponding cytosolic domain of the cation-independent-MPR (CI-MPR)^10^,^13^,^14^.

Adaptor proteins are implicated in the transport of sortilin and its cargo from the TGN to endosomes. The multimeric adaptor protein AP-1 and the monomeric gamma-ear-containing ADP ribosylation factor (ARF)-binding proteins (GGAs) interact with the cytosolic tail of sortilin^10^. AP-1/GGAs interactions with sortilin are essential for the transport of the receptor-ligand via clathrin-coated vesicles to endosomes^10^,^13^,^14^,^15^. Once the cargo is released, the retrograde transport of sortilin from endosomes to the TGN depends on its interaction with the Vps35 subunit of the retromer complex^13^ or palmitoylation in the cysteine 783 of its cytosolic tail^16^.

There is growing evidence that sortilin could have a function in both the innate and adaptive immune response. The trafficking of at least two lysosomal proteins is partially regulated by sortilin: prosaposin^17^ and acid sphingomyelinase (ASMase)^18^. Proteins of this family are antibacterial^19^,^20^, required for a selective degradation of membrane lipids^21^,^22^, phagosome maturation^23^, and antigen presentation^24^. Sortilin is up regulated in macrophages during the killing of mycobacteria^25^ and is required for the delivery of both prosaposin and ASMase from the Golgi complex to phagosomes in a route that is independent from the delivery of enzymes such as cathepsin D^26^. Sortilin has also been implicated in the secretion of IFN-γ and granzyme A by T and NK cells^27^. Sortilin is highly expressed in monocytes and macrophages^26^,^28^. However, the intracellular function of this sorting receptor in phagocytes during the immune response to infection with intracellular bacteria is not known.

Here, we investigated the function of sortilin in macrophages during infection with *M. tuberculosis*. We show that sortilin is acquired by mycobacteria-containing phagosomes via interactions with the adaptor proteins AP-1 and GGAs. The phagosomal association of sortilin is critical for the delivery of ASMase and required for efficient phagosome maturation. More importantly, macrophages lacking sortilin are not able to restrict the growth of both *M. bovis* BCG and *M. tuberculosis*. *In vivo*, *M. tuberculosis* burden was significantly higher in *Sort1*^−/−^ than in *Sort1*^+/+^ mice and *Sort1*^−/−^ animals showed an increase in lung infiltration of neutrophils and pathology. Altogether, our data indicate that sortilin is a new component of the phagosome maturation pathway and the killing machinery of macrophages and impacts on lung inflammation *in vivo*.

## Results

### Sortilin is recruited early into mycobacterial phagosomes

**Figure 1:**
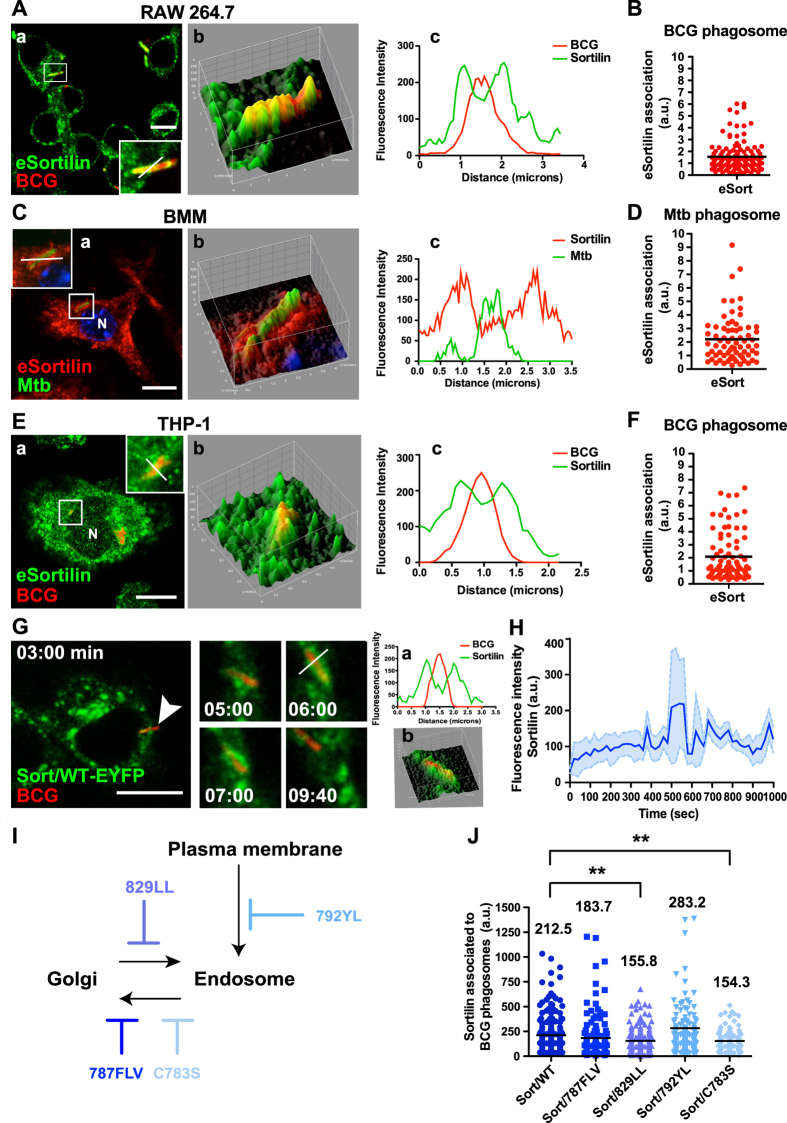
Sortilin is recruited early into mycobacterial phagosomes via GGAs/AP-1 and retromer interactions. (**A**) RAW 267.4 macrophages were infected with *M. bovis* BCG-DsRed (2 h uptake and 1 h chase) and stained for sortilin. Panel b: 3D-colocalization plot from inset in panel a. Panel c: profile of fluorescence intensity of a phagosome (white bar in inset in panel a). (**B**) Fluorescence intensity of endogenous sortilin associated with BCG phagosomes from 3 independent experiments. (**C**) BMM were infected with *M. tuberculosis*-EGFP (2 h uptake and 1 h chase) and stained for endogenous sortilin. Panel b: 3D-colocalization plot obtained from inset in panel a. Panel c: profile of fluorescence intensity of a representative phagosome (white bar in inset in panel a). (**D**) Fluorescence intensity of endogenous sortilin associated with *M. tuberculosis* phagosomes from two independent experiments. (**E**) THP-1 cells were infected with *M. bovis* BCG-DsRed (2 h uptake and 1 h chase) and stained for endogenous sortilin. Panel b: 3D-colocalization plot from inset in panel a. Panel c: profile of fluorescence intensity along the phagosome (white bar in inset in panel a). (**F**) Fluorescence intensity of endogenous sortilin associated with BCG phagosomes from two independent experiments. (**G**) Analysis by live cell imaging of association of Sort/WT-EYFP with phagosomes in RAW 267.4 macrophages. Panel a shows the profile of fluorescence intensity along the phagosome (white bar panel G). Panel b: 3D-colocalization plot from inset of panel G. (**H**) Quantitative analysis of the association of sortilin with BCG phagosomes (n = 5). The blue curve is the mean of the phagosomes analyzed and the light dashed blue curves represent the S.E.M. (**I**) Schematic representation of the sortilin trafficking affected by the mutations in the cytosolic tail. (**J**) RAW 267.4 macrophages expressing the indicated mutants were infected with BCG-DsRed (2 h uptake and 1 h chase), fixed and stained for sortilin. Scale bars: 10 μm. Data represents the Mean ± S.E.M of three independent experiments, at least 100 cells were analysed. (**)p ≤ 0.01 from one-way ANOVA with Tukey’s post hoc test.

Sortilin contributes to the delivery of specific lysosomal proteins from the Golgi complex to latex bead phagosomes^26^, but its function in mycobacteria-containing phagosome maturation is not known. We first analyzed if sortilin is recruited into mycobacteria-containing phagosomes. For that, macrophages were infected with *M. bovis* BCG–DsRed (BCG-DsRed) or *M. tuberculosis* H37Rv-EGFP (Mtb-EGFP) and sortilin was detected by indirect immunofluorescence. Quantitative analysis showed a strong association of endogenous sortilin with BCG-DsRed-containing phagosomes in RAW 264.7 macrophages (Fig. 1A,B). Endogenous sortilin was also associated with Mtb-EGFP-containing phagosomes in primary bone marrow-derived murine macrophages (BMM, Fig. 1C,D) and with BCG-DsRed-containing phagosomes in THP-1 human macrophages (Fig. 1E,F). The fluorescent intensity profiles along the phagosomes in the different types of macrophages were determined, confirming that sortilin was recruited to phagosomes containing *M. bovis* BCG and *M. tuberculosis*. By live cell imaging, we analyzed the kinetic of sortilin association with phagosomes at early time points (see material and methods). Sort/WT-EYFP was associated with BCG-DsRed-containing phagosomes after 1 min of internalization and remained associated with the phagosomes for at least 15 min (Fig. 1G,H, Movie-S1). After 15 minutes, Sort/WT-EYFP dynamically associated with phagosomes, but in transient interactions involving often kiss and run events (data not show).

### Sortilin is recruited by phagosomes via GGAs, AP-1 and retromer interactions

We then investigated the sorting motifs that mediate the delivery of sortilin to mycobacterial phagosomes (Fig. S2A). In macrophages, Sort/WT-EYFP localized in the Golgi complex and the endosomal fraction as reported before^26^. A mutant in the GGA/AP-1 binding motif that mediates anterograde transport from the TGN, Sort/829LL-EYFP was more pronounced associated with the cis-Golgi and TGN (Fig. S1), and less with endocytic structures (EEA-1 positive, Fig. S1). In contrast, two mutants in the residues C783S or 787FLV, implicated in the retromer-dependent endosome to TGN retrieval (Sort/C783S-EYFP and Sort/787FLV-EYFP) were less associated with the Golgi complex and more associated with endosomal structures (Fig. S1). As expected, a mutant in the endocytosis internalization motif Y792A/L795A (Sort/792YL-EYFP) remained associated with plasma membrane and association with the Golgi complex and endosomes was very low (Fig. S1).

Next, we analyzed the association of the different mutants to mycobacteria-containing phagosomes in fixed cells (see material and methods). Sort/WT-EYFP was associated with phagosomes (Mean = 212.5 ± 14.47). The association of the mutant in the anterograde transport Sort/829LL-EYFP with mycobacteria-containing phagosomes was significantly lower (Mean = 155.8 ± 9.771). Moreover, the association of the mutant in the retrograde transport Sort/C783S-EYFP with phagosomes was also reduced (Mean = 154.3 ± 10.64, Fig. 1J). The association of the Sort/787FLV-EYFP and Sort/792YL-EYFP mutants with phagosomes was not affected (Fig. 1J). These results indicate that the recruitment of sortilin into mycobacterial phagosomes was impaired in cells expressing mutants compromised in both anterograde and retrograde transport. Importantly, the internalization of the bacteria was not affected by the expression of the sortilin mutants (Fig. S2B) and there were not significant differences in the expression level of the different mutants in the transfected cells (Fig. S3). Thus, sortilin receptor or vesicles carrying sortilin are recruited early during internalization into mycobacteria-containing phagosomes and this association partially depends on interactions of sortilin with both GGAs/AP-1 and the retromer complex.

### Sortilin is required for phagosome maturation and restriction of mycobacterial growth in macrophages

**Figure 2:**
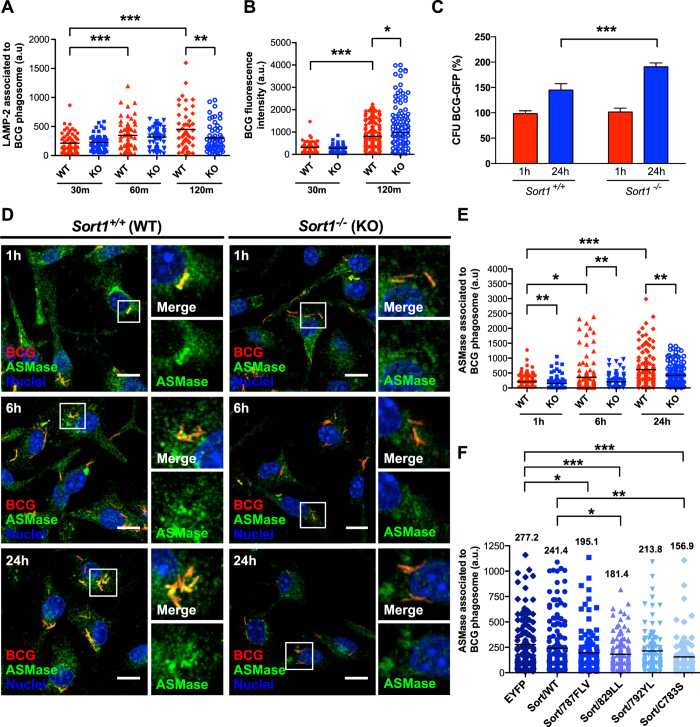
Sortilin is required for phagosome maturation and mycobacterial restriction growth. (**A**) BMM from *Sort1*^+/+^ (WT) or *Sort1*^−/−^ (KO) mice were infected with BCG-DsRed for 1 h of uptake, followed by 30 min, 1 h or 2 h of chase. Cells were fixed, stained for LAMP-2 and LAMP-2 association with BCG phagosomes analyzed. Data represent the Mean ± S.E.M of three independent experiments. At least 100 phagosomes were analyzed. (**B**) Quantitative analysis of the fluorescence signal intensity of BCG per cell after 30 min or 2 h of infection in BMM. Data represent the Mean ± S.E.M of three independent experiments. At least 100 cells were analyzed. (**C**) BMM from *Sort1*^+/+^ and *Sort1*^−/−^ mice and infected with BCG-DsRed for 1 or 24 h were lysed and CFU determined. Data represents the Mean ± S.E.M of three independent experiments. Two-tailed Student’s t-test was applied. (**D**) BMM from *Sort1*^+/+^ and *Sort1*^−/−^ mice were infected with BCG-DsRed (red) for 1, 6 or 24 h. Cells were fixed stained for ASMase (green). Scale bars: 10 μm. Insets show the association of ASMase to the BCG phagosomes. Nuclei were stained with Hoechst 33538 (blue). (**E**) Quantitative analysis of the ASMase association with BCG phagosomes in BMM from *Sort1*^+/+^ and *Sort1*^−/−^ mice after 1, 6 and 24 h of infection. Data represent the Mean ± S.E.M of three independent experiments. (**F**) RAW 264.7 macrophages expressing the indicated proteins were infected with BCG-DsRed for 2 h of uptake, followed by 1 h of chase and stained for ASMase. Quantitative analysis of the fluorescence signal of ASMase associated with BCG phagosomes. The numbers in the graph correspond to the mean of the fluorescence signal of ASMase (a.u = arbitrary units). Data represent the Mean ± S.E.M of three independent experiments. At least 100 phagosomes were analyzed. (*)p ≤ 0.05, (**)p ≤ 0.01, (***)p ≤ 0.001 from one-way ANOVA with Tukey’s post hoc test.

Sortilin is transcriptionally up-regulated during the process of mycobacterial killing in macrophages^25^, thus we sought to analyze the role of sortilin during this process. We observed a significant decrease in the number of mycobacterial phagosomes positive for LAMP-2 in bone marrow macrophages (BMM) from *Sort1*^−/−^ mice compared to mycobacterial phagosomes in *Sort1*^+/+^ BMM (Mean = 450.5 ± 47.29 vs. 303.4 ± 31.83, respectively) after 2 h of infection (Figs 2A and S4A). No differences were observed in the percentage of infected cells at the different time points (Fig. S4B). However, the total fluorescence intensity of BCG-DsRed per cell increased in BMM from *Sort1*^−/−^ mice after 2 h of infection compared to BMM from *Sort1*^+/+^ (Fig. 2B). Next, we evaluated the intracellular survival of mycobacteria in BMM from *Sort1*^+/+^ and *Sort1*^−/−^ mice by counting colony-forming units (CFU). After 24 h of infection, the total CFU of mycobacteria was 190.77% higher in *Sort1*^−/−^ macrophages than in *Sort1*^+/+^ macrophages (Fig. 2C). We concluded that sortilin is required for phagosome maturation and more importantly for mycobacterial growth restriction.

### Sortilin mediates ASMase delivery into mycobacterial phagosomes via interactions with GGAs, AP-1 and retromer

Sortilin regulates a pathway that delivers ASMase to latex-bead phagosomes^26^. Considering that ASMase plays an important role in the biological function of macrophages and more importantly, in the regulation of phagosome maturation^23^,^29^, we investigated if ASMase was present in mycobacterial phagosomes. The expression of the different mutants caused a redistribution of ASMase that was retained in the Golgi complex in cells expressing the anterograde mutant and in endocytic structures in cells expressing the retrograde mutants (Fig. S5). Distribution of ASMase was not affected in cells expressing the internalization mutant Sort/792YL-EYFP (Fig. S5). BMMs from *Sort1*^+/+^ or *Sort1*^−/−^ mice were infected and the association of ASMase with phagosomes was analyzed. The distribution of ASMase was similar in both *Sort1*^+/+^ and *Sort1*^−/−^ macrophages (Fig. 2D). However, a quantitative analysis showed that the association of ASMase was significantly higher in mycobacteria-containing phagosomes from *Sort1*^+/+^ compared to *Sort1*^−/−^ BMM at 1, 6 and 24 h of infection (Fig. 2E). These data indicated that sortilin primarily transports ASMase into mycobacterial-containing phagosomes. The expression of the mutant of sortilin with a deletion of the C-terminal dileucine motif (Sort/829LL-EYFP, mutant in the anterograde pathway, Mean = 181.4 ± 13.57) and the mutants in the residues affecting the interaction of sortilin with the retromer complex (Sort/C783S-EYFP and Sort/787FLV-EYFP, mutants in retrograde pathway, Mean = 156.9 ± 16.33 and 195.1 ± 21.22 respectively) impaired the association of ASMase with mycobacteria-containing phagosomes (Figs 2F and S6). As expected, the mutant in the internalization motif Sort/792YL did not affect ASMase delivery into the phagosome. Taken together, our results indicated that the delivery of ASMase into mycobacterial phagosomes depends on sortilin and its ability to interact with components of GGA/AP-1-mediated anterograde and retromer-mediated retrograde pathways.

### Sortilin-dependent phagosome maturation and mycobacterial restriction of growth requires interactions of sortilin with GGAs, AP-1 and the retromer complex

**Figure 3:**
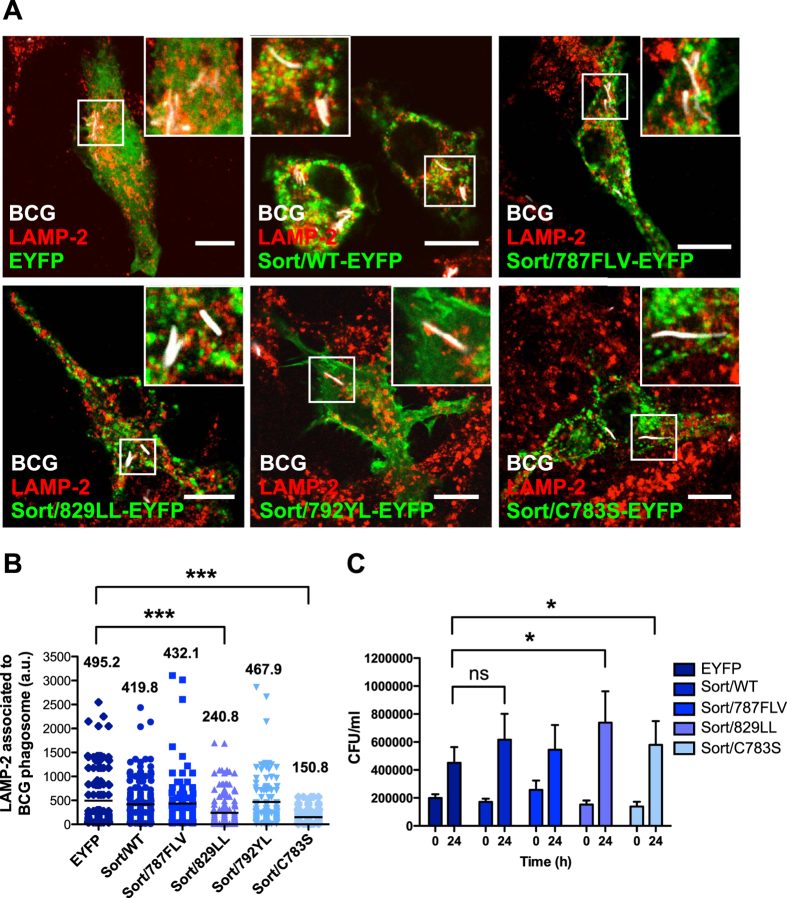
The interaction of sortilin with GGAs/AP-1 and the retromer complex is required for phagosome maturation and mycobacterial restriction of growth. (**A**) RAW 267.4 cells expressing the indicated proteins and infected with BCG-DsRed (white) for 2 h of uptake, followed by 1 h of chase were fixed and stained for LAMP-2 (red). Scale bar: 10 μm. (**B**) Quantitative analysis of fluorescence signal intensity for LAMP-2 association with BCG phagosomes. The numbers in the graph correspond to the mean of the fluorescence signal of LAMP-2 associated to BCG phagosome (a.u = arbitrary units) in the transfected cells. Data represents the Mean ± S.E.M of four independent experiments. At least 150 phagosomes were analyzed. (***)p ≤ 0.001 from one-way ANOVA with Tukey’s post hoc test. (**C**) Macrophages expressing the different constructs were infected with BCG-DsRed at the indicated time points. Cells were lysed and CFU determined. Data represent the Mean ± S.E.M of the CFU recovered compared with control cells (expressing EYFP) from three independent experiments. ns = not significant p > 0.01(*), (*)p ≤ 0.05 from two-tailed Student’s t-test.

To understand the molecular mechanisms underlying the role of sortilin in the maturation of mycobacterial phagosomes, we sought to identify the sorting motif(s) that might have an effect in phagosome maturation. For this, macrophages expressing different mutants in the sortilin cytosolic tail were infected with mycobacteria and labeled for LAMP-2. Notably, the association of LAMP-2 with mycobacteria-containing phagosomes was reduced in cells expressing the mutant deficient in both GGAs/AP-1 and retromer complex interactions. More than 50% of reduction was observed in cells expressing the mutant Sort/829LL-EYFP and Sort/C783S-EYFP compared to EYFP and Sort/WT-EYFP (Fig. 3A,B). This was not due to a general re-localization of LAMP-2 positive compartments (Fig. 3A). We next tested whether the impaired anterograde or retrograde trafficking elicited by the expression of sortilin mutants had an effect in mycobacterial survival. As shown in Fig. 3C, the survival of the bacteria increased in macrophages overexpressing Sort/829LL-EYFP and Sort/C783S-EYFP compared to cells expressing EYFP, suggesting that a normal transport of sortilin in macrophages is required for phagosome maturation and efficient restriction of mycobacterial growth.

### Sortilin regulates *M. tuberculosis* phagosome maturation and growth restriction in macrophages

**Figure 4:**
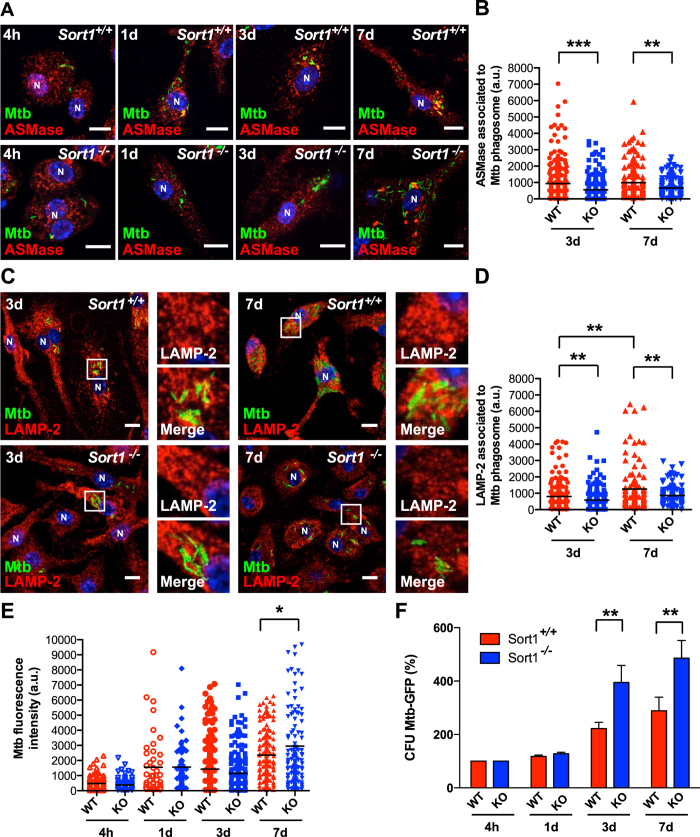
Sortilin regulates *M. tuberculosis* phagosome maturation and growth restriction in macrophages. (**A**) BMM from *Sort1*^+/+^ (WT) and *Sort1*^−/−^ (KO) mice were infected with *M. tuberculosis*-EGFP for 1 h of uptake, followed by 4 h, 1, 3 and 7 days of chase. Cells were fixed and stained for ASMase (red). Nuclei displayed in blue (N). Scale bar: 10 μm. (**B**) Analysis of the fluorescence signal intensity of ASMase associated with *M. tuberculosis* phagosomes in BMM from *Sort1*^+/+^ and *Sort1*^−/−^ mice after 3 and 7 days of infection. Data represent the Mean ± S.E.M of three independent experiments. (**C**) *Sort1*^+/+^ and *Sort1*^−/−^ BMMs infected with *M. tuberculosis*-EGFP for 1 h of uptake, followed by 3 and 7 days of chase were fixed and stained for LAMP-2 (red). Nuclei displayed in blue (N). Scale bar: 10 μm. (**D**) Analysis of the fluorescence signal intensity of LAMP-2 associated with *M. tuberculosis* phagosomes in BMM from *Sort1*^+/+^ and *Sort1*^−/−^ mice after 3 and 7 days of infection. Data represent the Mean ± S.E.M of three independent experiments. (**E**) Quantitative analysis of the fluorescence signal intensity of *M. tuberculosis*-EGFP in BMMs from *Sort1*^+/+^ and *Sort1*^−/−^ mice at different post-infection times. Data represent the Mean ± S.E.M of three independent experiments. (**F**) BMM from *Sort1*^+/+^ and *Sort1*^−/−^ mice were infected with *M. tuberculosis*-EGFP and at the indicated time points were lysed and CFU determined. Data represent the Mean ± S.E.M of three independent experiments. In all the panels (*)p ≤ 0.05, (**)p ≤ 0.01, (***)p ≤ 0.001 from one-way ANOVA with Tukey’s post hoc test or from two-tailed Student’s t-test.

Because sortilin was required for the maturation of BCG-containing phagosomes, we tested whether the sortilin-dependent phagosome maturation was required for growth control of the pathogenic strain *M. tuberculosis* H37Rv. BMM from *Sort1*^+/+^ and *Sort1*^−/−^ mice were infected with Mtb-EGFP at different time points, fixed and the association of ASMase and LAMP-2 with *M. tuberculosis*-containing phagosomes was examined by fluorescence microscopy. A reduction of ASMase association with *M. tuberculosis*-containing phagosomes occurred in *Sort1*^−/−^ compared to *Sort1*^+/+^ BMM after 3 and 7 days of infection (Fig. 4A,B). Consistent with previous data (Fig. 2A), the accumulation of the late endosomal marker LAMP-2 in *Sort1*^−/−^ BMM significantly decreased (Fig. 4C,D). Moreover, treatment of macrophages with desipramine, to deplete ASMase^30^ increased the survival of *M. tuberculosis* (Fig. S7), indicating that in mouse macrophages, ASMase is required for *M. tuberculosis* growth restriction. Next, we assessed whether sortilin had an impact on *M. tuberculosis* infection. BMM from *Sort1*^−/−^ mice showed a significant increase in the *M. tuberculosis*-EGFP fluorescence intensity signal at the single cell level compared with *Sort1*^+/+^ macrophages after 7 days of infection (Fig. 4E). However, this parameter was not affected at early time of infection, indicating that the internalization of the bacteria was not affected. After 3 and 7 days of infection, a significant increase in the survival of *M. tuberculosis* was observed in *Sort1*^−/−^ macrophages compared to *Sort1*^+/+^, as determined by CFU analysis (Fig. 4F). Taken together, our data show that sortilin regulates both the trafficking of ASMase to *M. tuberculosis* phagosomes and *M. tuberculosis* growth restriction in macrophages.

### *In vivo* role of sortilin in the mouse model of tuberculosis

**Figure 5:**
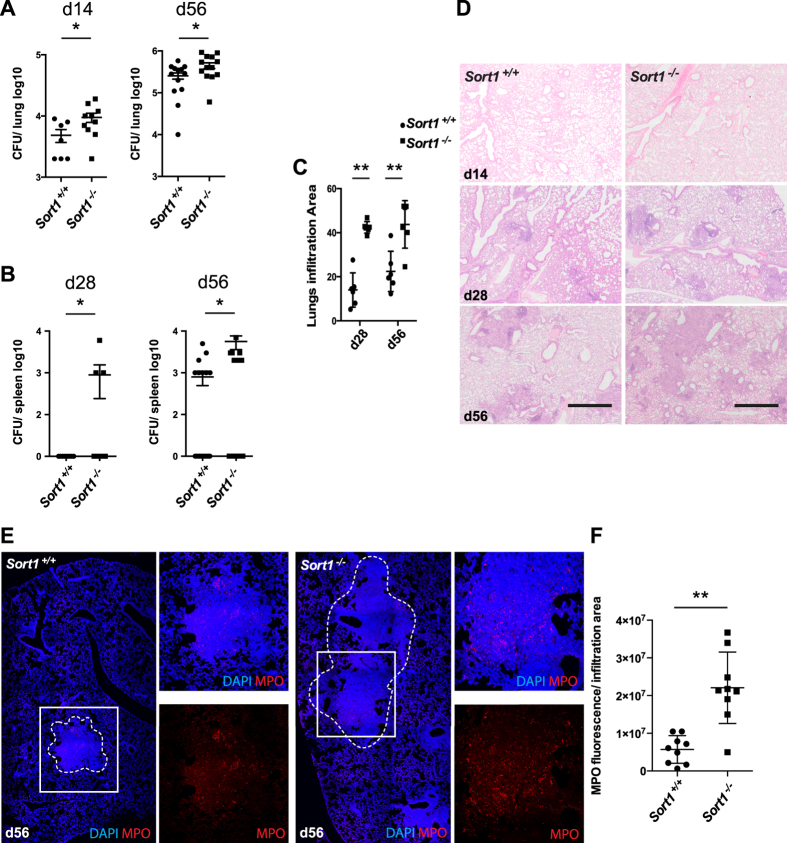
*In vivo* role of sortilin in mycobacterial infection. (**A)** Growth of *M. tuberculosis* in lungs of *Sort1*^+/+^ and *Sort1*^−/−^ mice following exposure to a low dose infection. (*)p ≤ 0.05 from two-tailed Student’s t-test. (**B**) Growth of *M. tuberculosis* in spleens of *Sort1*^+/+^ and *Sort1*^−/−^ mice after 28 and 56 days of infection. (*)p ≤ 0.05 from two-tailed Student’s t-test. (**C**) Quantitation of the infiltrated area in infected lungs as indicated in material and methods, (**)p ≤ 0.01, from two-tailed Student’s t-test. (**D**) Representative histological sections stained with hematoxylin and eosin of *M. tuberculosis* infected lungs in *Sort1*^+/+^ and *Sort1*^−/−^ mice after the indicated times of infection. Scale bar: 0.5 μm. (**E**) Neutrophil recruitment in lesions of *M. tuberculosis* infected mice. Scale bar: 0.5 μm. (**F**) Quantitative analysis of MPO fluorescence/area in three independent experiments (**)p ≤ 0.01 from two-tailed Student’s t-test.

To address the role of sortilin in susceptibility to tuberculosis *in vivo*, we infected *Sort1*^−/−^ and *Sort1*^+/+^ mice with *M. tuberculosis* H37Rv. Using a low dose infection, mimicking conditions associated with natural aerosol transmission (CFU between 100–200), we identified a modest but significant difference in bacterial load among *Sort1*^−/−^ and *Sort1*^+/+^ mice after 14 and 56 days post-aerosol challenge (Fig. 5A). Additionally, we observed more rapid dissemination into spleens after 28 and 56 days of infection in *Sort1*^−/−^ mice (Fig. 5B). More importantly, we observed a significant increase in the size of infiltrated areas in the lungs of *Sort1*^−/−^ mice after different infection times (Fig. 5C,D). This increased pathology correlated with a significantly higher recruitment of neutrophils into the lesions in *Sort1*^−/−^ mice (Fig. 5E,F). Altogether, our results suggest that sortilin is required to limit bacterial replication by macrophages, and lack of this receptor leads to lung cellular infiltration, neutrophil recruitment and immunopathology in the mouse model of tuberculosis infection.

## Discussion

To our knowledge, this is the first time that a lysosomal receptor deficiency is associated with susceptibility to tuberculosis in the mouse model of infection. Using different macrophages and mycobacteria we demonstrated here a role for the intracellular pool of sortilin in macrophages and in the innate immune response. We previously reported that sortilin is associated with latex bead-containing phagosomes and this association depends on its cytosolic tail^26^. Here we demonstrated that both GGAs/AP-1 interaction and palmitoylation of the cysteine in the cytosolic tail (necessary for binding to retromer complex) were required for the delivery of sortilin to mycobacterial phagosomes (See model Fig. S8). The cytosolic tail of the sortilin receptor contains sorting signals, which mediate transport from the TGN to endosomes and, after cargo release, the retrieval from endosomes^14^,^31^. We observed that sortilin delivery into mycobacterial phagosomes depends on the sortilin interaction with both GGA/AP-1 and the retromer complex. GGA/AP-1 complex is essential for the anterograde trafficking of sortilin^10^,^13^,^15^ and our data indicate that this step is also required for transport to phagosomes. Moreover, palmitoylation of sortilin in the cysteine 783 of its cytosolic tail is necessary for the retrograde trafficking of sortilin via the retromer complex from endosomes to the Golgi complex^16^. The precise mechanism by which interaction of sortilin with the retromer regulates phagosome maturation and cargo delivery into phagosomes remains to be identified. Interestingly, there is increasing evidence of the contribution of the retromer to phagosome maturation^32^,^33^. For example, the *Legionella pneumophila* Icm/Dot-translocated effector protein RidL is an interacting protein of the retromer complex. This bacterial protein blocks retrograde transport and promotes intracellular replication^34^.

In order to effectively degrade phagocytosed bacteria within phagolysosomes, hydrolytic enzymes are essential^1^. We have identified here that sortilin regulates the transport of ASMase to mycobacteria-containing phagosomes and this requires an efficient sortilin trafficking. Although there are likely other enzymes delivered by sortilin into phagosomes, our results are in line with the notion that ASMase is important for the killing of *Salmonella enterica* serovar Typhimurium^35^ and *Listeria monocytogenes*^23^,^36^. In another scenario, ASMase could hydrolyze the phagosomal lipid sphingomyelin into ceramide and phosphorylcholine, enhancing the fusogenicity of cellular membranes^36^. Thus, it is likely that a decrease of ASMase will affect mycobacterial killing capacity in the phagosomal compartment by impairing fusion of phagosomes with lysosomes. On the other hand, ASMase contributes to macrophage necrosis in the presence of and excess of TNF-α, through the activation of the ASMase that results in the production of ceramide, an inducer of apoptosis and necrosis. The knockdown of ASMase in the zebrafish model reduced ceramide production and prevented macrophage necroptosis, increasing macrophage microbicidal activity and reducing the *M. marinum* burdens. Ceramide production occurs in the context of high TNF-α production^30^. In the light of our results, it is clear that ASMase can have multiple roles during bacterial infections as previously postulated^36^. On one hand, ASMase could be linked to bacterial infections by modulating host cell death^30^. On the other hand, ASMase can also affect lysosomal activities or fusogenic properties of intracellular vesicles. Consistent with this notion, ceramide can facilitate killing of pathogenic mycobacteria by promoting the maturation of phagosomes hitherto arrested in the early phagosomal stage^37^. In agreement with this hypothesis, we also found that sortilin-mediated trafficking modulates phagosome maturation via LAMP-2 accumulation in mycobacterial phagosomes. However, the complete lack of ASMase results in macrophages to become “foamy”, a niche where mycobacteria grow^29^,^38^.

The differences in bacterial killing are unlikely to be due to a defect in a general lysosomal function such as a primary defect in critical lysosomal enzymes in late endosomes/lysosomes, as observed in M6PR deficient cells^39^. The knockout of M6PR in mice is lethal whereas sortilin knockout mice are viable^40^. This suggests that sortilin regulates a more specific lysosomal-related function in immune cells rather than a general lysosomal function. Moreover, our data is consistent with sortilin being part of a previously reported direct pathway from the Golgi to phagosomes^26^,^41^,^42^,^43^, that delivers specific proteins required for *M. tuberculosis* control in macrophages.

Previous reports indicated that cytotoxic T cells and NK cells from *Sort1*^−/−^ mice appear to have reduced secretion of IFN-γ in cytotoxic T lymphocytes impairing clearance of adenoviruses and increasing *Listeria monocytogenes* numbers in the spleen of infected mice compared to *Sort1*^+/+^^27^. We expected a more dramatic effect in *M. tuberculosis* infected mice given that IFN-γ is a key cytokine for the control of tuberculosis^44^. However, although there is a difference in IFN-γ release between *Sort1*^+/+^ and *Sort1*^−/−^ mice, there is still significant production of IFN-γ and that is likely sufficient for control of *M. tuberculosis*^27^. In addition, we did not observe significant IFN-γ release from splenic or lymph node lymphocytes in *Sort1*^−/−^ mice after mycobacterial infection (Fig. S9). Our findings suggest a model where the absence of sortilin in macrophages leads to uncontrolled bacterial replication in the lung, which causes increased pathology and neutrophil recruitment into the lesions. Our data also suggest that *in vivo*, early macrophage control of bacterial growth prevents dissemination of the infection from the lungs to secondary sites. Alternatively, sortilin might have a function in the production of mediators of inflammation and the precise differences and identity of these immune factors remain to be identified. However, during neuroinflammation, sortilin primarily plays a role in the innate rather than adaptive immune response^28^. Supporting this idea, *M. tuberculosis*-infected animals show a more severe defect in the innate response and do not succumbs to infection likely due to the development of an adequate adaptive immune response.

Taken together, our results uncover a novel sortilin-dependent pathway by which specific factors such as ASMase and likely other unidentified factors are transported into mycobacterial phagosomes. Association of sortilin with phagosomes leads to phagosome maturation, mycobacterial control and inflammation *in vivo*.

## Experimental Procedures

### Cells and mycobacteria

RAW 264.7 macrophages were obtained from the American Type Culture Collection (ATCC, Cat# TIB-71) and maintained in complete Dulbecco’s Modified Eagles Medium (D-MEM) with 4.5 g/L glucose, 10% (v/v) heat inactivated fetal calf serum (FCS, PAA, Austria) and 2 mM L-glutamine (PAA, Austria) (full D-MEM medium). Bone marrow macrophages (BMMs) were isolated and maintained as described previously^45^. Cells were incubated at 37 °C and 5% CO_2_ in a humidified incubator. *M. bovis* bacillus Calmette–Guérin (BCG) str. Pasteur 1173P2 expressing Ds-Red was kindly provided by Dr. Brigitte Gicquel (Pasteur Institute, France). *M. tuberculosis* H37Rv was kindly provided by Dr. Douglas Young (The Francis Crick Institute, UK). EGFP-expressing *M. tuberculosis* was generated transforming *M. tuberculosis* with pML1335^46^. Mycobacteria were grown in roller flasks at 37 °C in Middlebrook 7H9 liquid medium (Difco Laboratories, USA) containing 0.2% glycerol, 0.05% Tween-80 and supplemented with 10% oleic acid-albumin-dextrose-catalase supplement (OADC, BD Biosciences, USA) or in Middlebrook 7H10 plates (Difco Laboratories, USA) supplemented with 10% OADC. Sortilin wild type and mutants expression vectors were previously described^47^.

### Infection of macrophages with mycobacteria

RAW 267.4 macrophages or BMM from *Sort1*^+/+^ and *Sort1*^−/−^ mice were infected with *M. bovis* BCG-DsRed or *M. tuberculosis*-EGFP in full D-MEM medium. A single-bacteria suspension at optical density (OD) 600 nm = 0.025 was added for 2 h of uptake. After cells were washed 3 times with PBS, DMEM medium was added and incubated at different time points. In some experiments, 25 μM desipramine (Sigma, USA) was added to induce ASMase degradation (Fig. S7). For THP-1 infections, THP-1 cells were obtained from the American Type Culture Collection (ATCC) and maintained in complete RPMI Medium with 4.5 g/L glucose, 10% (v/v) heat inactivated fetal calf serum (FCS, PAA, Austria) and 2 mM L-glutamine (PAA, Austria) (full RPMI medium). The day before infection, cells were plated in full RPMI medium plus 50 nM of PMA to allow differentiation. The day of infection, cells were washed and a suspension of *M. bovis* BCG-DsRed in full RPMI medium with optical density (OD) 600 nm = 0.025 was added for 2 h of uptake and 1 h of chase. Next, cells were fixed and subjected to immunofluorescence to detect endogenous sortilin using specific antibodies.

### Macrophage transfection

Transfection of RAW 264.7 macrophages was performed using Lipofectamine2000 (Invitrogen, Germany). Briefly, cells were seeded in plates 1 day before the transfection. The day of transfection, cells were washed with PBS and then incubated with the transfection mix for 6 h in OptiMEM cell culture medium (Invitrogen, Germany). After incubation, fresh medium containing serum was added. Cells were subsequently used for further studies.

### Indirect immunofluorescence

Cells were processed as described before^26^ and in supplementary material using a Leica SP5 confocal microscope (Leica Microsystems). The primary and secondary antibodies used in this work were obtained from the same batches and always utilized at the same dilutions. The following antibodies were used: rabbit anti-ASMase (Santa Cruz Biotechnologies, USA) with a dilution of 1:50 overnight in humid chamber at room temperature, rat anti-LAMP-2 (Hybridoma Bank, Iowa, USA) with a dilution of 1:50 during 2 h at room temperature, rabbit polyclonal antibody anti-sortilin (kindly provided by Dr. Claus Petersen, University of Aarhus, Denmark) with a dilution of 1:50 over night in humid chamber at room temperature. Mouse anti-EEA1 (BD Bioscience) with a dilution of 1:50 over night in humid chamber at room temperature, mouse anti-GM130 (BD Bioscience) used with a dilution 1:100, 2 h at room temperature and mouse anti-Syntaxin6 (BD Bioscience) with a dilution of 1:50 over night in humid chamber at room temperature. Secondary antibodies used for indirect immunofluorescence were: goat anti-rabbit Alexa633 (dilution 1:600), goat anti-mouse Alexa633 (dilution 1:600), goat anti-rabbit Alexa488 (dilution 1:600) and goat anti-rabbit Alexa546 (dilution 1:600) (Invitrogen, USA). Secondary antibodies goat anti-Rat Cy3 and goat anti-Rat Cy5 were from Jackson Research Inc, USA.

### Colony-forming units (CFU) analysis

Mycobacteria were used to infect RAW 267.4 cells or BMMs (MOI for BCG: 2.5 and for H37Rv: 1). After 2 h of uptake, cells were washed 3 times with PBS and fresh medium plus gentamicin 10 μg/mL (Sigma, Germany) was added. Cells were incubated at the indicated time points at 37 °C in 5% CO_2_ atmosphere. Subsequently, cells were lysed in sterile water and serially diluted in PBS- Tween-80 0.05%. Dilutions were plated in Middlebrook 7H10 complete agar medium (Difco Laboratories, Germany). Colonies were counted after 3 to 4 weeks to determine CFU.

### Mouse infections

Balb/C (WT) and *Sort1*^−/−^ mice^40^ were bred and housed under specific-pathogen-free conditions at the Francis Crick Institute, Mill Hill Laboratory. All animals were bred and maintained for experiments in accordance with the United Kingdom Home Office regulations. All experimental protocols were approved by the United Kingdom Home Office (project license 7008045). Groups of female 6–8 week old *Sort1*^−/−^ and Balb/C wild-type control mice were infected by low-dose aerosol exposure with a growing (mid log phase) culture of H37Rv *M. tuberculosis* using a Glas-Col (Terre Haute, IN) aerosol generator calibrated to deliver approximately 100 bacteria into the lungs. Bacterial counts in the lungs and spleen (n = 5) at each time point of the study were determined by plating serial dilutions of individual organ homogenates on duplicate plates of Middlebrook 7H10 agar containing OADC enrichment. CFU were counted after 3–4 weeks incubation at 37 °C.

### Histology and measurement of infiltration

Separate whole lungs samples were fixed by inflating the tissues with formaldehyde. Formalin-fixed lungs were embedded, sectioned, and stained by hematoxylin and eosin (H&E) at the Laboratory of Histology at the Francis Crick Institute. Slides were examined in an Olympus VS120 Slide reader microscope equipped with a colour digital camera. To determine the lung area covered by cellular infiltration, the ImageJ software program was used to calculate the area of immune infiltration. Percentage of the total lung area covered by cellular infiltration was calculated for each mouse by dividing the sum of granulomatous areas in these sections by the total area of the lung examined. 5 mice per condition were used and 3 lungs sections per mouse were analysed from 3 different experiments. For neutrophil staining, tissue sections were deparaffinized in xylene (2 × 10 min, 100%, 95% and 80% ethanol 2 min each). Tissue sections were then placed into an antigen retrieval buffer (Access super antigen solution, Menarini diagnostics, UK) in a decloaking chamber (Biocare Medical, CA, USA); incubated at 110 degrees for 10 min and allowed to cool for 60 min. Sections were permeabilized in PBS-0.2% Triton X-100 and incubated in blocking buffer (1% BSA, 5% Fetal Calf Serum in PBS) overnight at room temperature. Polyclonal rabbit anti myeloperoxidase (MPO) antibody was from Dako (Dako, Denmark).

### Live cell imaging

1 × 10^5^ of transfected RAW 267.4 cells were seeded on 35 mm glass bottom dishes (MatTek, USA). Cells were washed with PBS and replaced with imaging medium containing *M. bovis* BCG-DsRed at optical density (OD) 600 nm = 0.05 in full D-MEM medium without phenol Red, shortly before imaging with a Leica SP5 AOBS Laser Scanning Confocal Microscope (Leica Microsystems, Germany) equipped with an environment control chamber (EMBLEM, Germany). For live cell imaging the settings were scanning mode xyzt, pixel resolution of 1,024 × 1,024, scanner frequency of 200 Hz, line averaging of 3, and zoom of 2. Frames were acquired every 20 seconds during 17 min. The time-lapse images were exported to uncompressed AVI-formatted movies as single channels with the LAS AF software (Leica microsystems). The RGB-color movies were loaded into Fiji (distribution of ImageJ available at http://fiji.sc., iterative versions of ImageJ used for this work are 1.41m through 1.46a) and transformed into 8-bit color movies. The 8-bit color movies were then ready for further quantification. To quantify the fluorescence of sortilin associated with the mycobacterial phagosome, the corresponding fluorescent-channel movies were loaded into Fiji. A region of interest (ROI), enclosing the bacteria in the red channel, was drawn by using the ‘polygon selections’ tool. In “Set Measurements” tool, only “Area” and “Integrated Density” were selected. Subsequently, the integrated intensity inside the ROI selected and redirected to the green channel (sortilin) was measured (“Analyze-Measure”). The position and the size of the ROI were adjusted manually with the movement of the bacteria in the different frames. Fluorescence intensity values were plotted and analyzed using Microsoft Excel 2011 (Microsoft) and GraphPad Prism 5 (GraphPad Software Inc., USA).

### Image analysis

Leica SP5 AOBS Laser Scanning Confocal Microscope was used (Leica Microsystems, Germany). During image acquisition for fixed samples, a single focal plane was monitored in time (xyt scanning mode) using a 63x/1.4 HCX-PLAPO oil objective, an Argon Laser (488 nm) and DPSS Laser (561 nm) when applicable, scanner frequency 200–400 Hz; line averaging 6, using PMT and/or HyD detectors at a scanning resolution of 1024 × 1024 pixels. Zoom of 2.5. The same settings of the laser powers, gain and offset were maintained for the different experiments. The software of the microscope, LAS AF, allows saving the exact settings to acquire the images used in the experiments; therefore with every experiment the corresponding settings were loaded.

Analyses of all the images were performed using ImageJ (U.S. National Institute of Health, Bethesda, Maryland, USA) and Fiji. To measure intracellular bacterial intensity, Leica SP5 files (.lif) were opened using Fiji and split into their constitutive color channels. The corresponding channel was subjected to a pixel threshold (the same threshold was selected in all the cases). The bacteria per cell were selected using “wand-tracing tool”, and the “Integrated Density” measurement was taken (the sum of the values of the pixels in the selection that correspond with the number of intracellular bacteria). To measure the association of a marker (e.g., primary antibodies against ASMase or LAMP-2) with bacterial particles in the cell, the RGB image was split into individual channels, and the red channel (bacteria) was subjected to a pixel threshold. The “wand-tracing tool” was used to select all the bacteria per cell, and then the “Analyze-Measure” function of Fiji was used to measure the fluorescence intensity of the marker of interest (corresponding to the secondary antibody used to detect the primary antibody) associated to the bacterial phagosome by re-directing the measurements to the channel of interest in “Set Measurements” in Analyses function of Fiji. All the images were acquired with the same zoom and the different experiments were combined all together. Fluorescence intensity values were plotted and analyzed using Microsoft Excel 2011 (Microsoft) and GraphPad Prism 5 (GraphPad Software Inc., USA). The plots corresponding to “3D-colocalization” and “fluorescence intensity profile” of representative phagosomes showed in Fig. 1 were performed using ImageJ and Fiji.

### Statistical analysis

Statistical calculations and normalizations were performed using GraphPad Prism software version 5.0a (GraphPad Software Inc., USA). Results are represented as the Mean ± S.E.M from at least three independent experiments. P-values were calculated using Student’s two-tailed t-test or one-way analysis of variance (ANOVA) as indicated.

## Additional Information

**How to cite this article**: Vázquez, C. L. *et al*. The proneurotrophin receptor sortilin is required for *Mycobacterium tuberculosis* control by macrophages. *Sci. Rep*. **6**, 29332; doi: 10.1038/srep29332 (2016).

## Supplementary Material

Supplementary Information

Supplementary Movie S1
