# Supplementary material for: The proneurotrophin receptor sortilin is required for Mycobacterium tuberculosis control by macrophages
Source: Sci Rep. 2016 Jul 8;6:29332. doi: 10.1038/srep29332 (PMC4937236; doi:10.1038/srep29332)
Supplement: Supplementary Information [file srep29332-s1.pdf]

Supplementary information

The proneurotrophin receptor sortilin is required for *Mycobacterium tuberculosis* control by macrophages

Cristina L. Vazquez, Angela Rodgers, Susanne Herbst, Stephen Coade, Achim Gronow, Carlos A. Guzman, Mark S. Wilson, Makoto Kanzaki, Anders Nykjaer and Maximiliano G. Gutierrez

Supplementary figures

Figure S1- Localization of sortilin wild type and mutants in macrophages. RAW 267.4 cells were transfected with the different sortilin constructs and after 24 h, cells were immune-stained using the antibodies anti-GM130 (Golgi complex), anti-Syntaxin6 (TGN) and anti-EEA1 (early endosome) to detect colocalization of sortilin with the different markers. Representative images showing the association of sortilin with GM130 (panels in the left), Stx6 (panels in the middle) and with EEA1 (panels in the right). Arrowheads show the association of sortilin with the different markers. Scale bar: 10 μm.

Figure S2-Internalization of mycobacteria in cells expressing sortilin wild type and mutants.

RAW 267.4 cells expressing the indicated constructs were infected with BCG-DsRed during 2 h to allow the internalization and 1 h of chase. Graph shows the quantitative analysis of the fluorescence signal intensity of BCG per cell. Data represent the Mean ± S.E.M of three independent experiments. At least 100 phagosomes were analyzed.

Figure S3- Expression levels of sortilin wild type and mutants in macrophages.

A- Images show Western blot analysis from cell lysates of macrophages expressing the different constructs. Expression level of sortilin was detected using anti-EGFP antibody and the loading control was tested using anti-tubulin antibody. **B-** Quantification of the expression level of sortilin in the different constructs by the analysis of the bands using Fiji software. Data represents the

Mean ± S.E.M of three independent experiments. N.s= no significant differences (ns) from one-way ANOVA with Tukey's post hoc test.

Figure S4- Distribution of LAMP-2 in *Sort1*$^{+/+}$ (WT) or *Sort1*$^{-/-}$ (KO) macrophages.

A- BMM from *Sort1*$^{+/+}$ (WT) or *Sort1*$^{-/-}$ (KO) mice were infected with BCG-DsRed (red) for 1 h of uptake and 30 minutes, 1 h or 2 h of chase. Cells were fixed and immune-stained for LAMP-2 (green). Nuclei showed in blue. Scale bar: 10 µm. **B-** Percentage of infection in BMM from *Sort1*$^{+/+}$ (WT) or *Sort1*$^{-/-}$ (KO) mice infected with BCG-DsRed for 1 h of uptake and 1 h, 2 h or 24 h chase. Data represents the Mean ± S.E.M from 3 independent experiments. N.s= no significant differences (ns) from one-way ANOVA with Tukey's post hoc test.

Figure S5- Distribution of ASMase in macrophages expressing sortilin wild type and mutants.

RAW 267.4 cells were transfected with the sortilin expression vectors. After 24 h, cells were stained for ASMase and analyzed by confocal microscopy. Representative images showing the association of sortilin (green) with ASMase (red). Scale bar: 10 µm. Insets show the colocalization (white) of both sortilin and ASMase in the transfected cells using the colocalization highlighter from Fiji.

Figure S6- Association of ASMase with mycobacteria-containing phagosomes.

RAW 267.4 cells transfected with the indicated vectors expressing different mutants in the cytosolic tail of sortilin were infected with BCG-DsRed for 2 h of uptake and 1 h of chase, fixed and stained for ASMase using a specific antibody (blue) and observed with a confocal microscope. Scale bar: 10 µm.

Figure S7- *M. tuberculosis* growth in desipramine-treated macrophages. BMM were infected with *M. tuberculosis*-EGFP in absence or presence of 25 µM desipramine during the chase period. Cells were lysed and CFU determined. Data represent the Mean ± S.E.M of two independent experiments. (*) $p \leq 0.05$, (**)

$p \leq 0.01$ from one-way ANOVA with Tukey's post hoc test or from two-tailed Student's t-test

Figure S8- Schematic representation of the role of sortilin in mycobacteria-containing phagosome maturation.

Once mycobacteria are internalized in macrophages, sortilin is recruited to the bacterial phagosome promoting its maturation by the acquisition of ASMase and lysosomal proteins like LAMP-2, a process that requires the interaction of sortilin with both GGAs/AP-1 and the retromer complex. Therefore, sortilin-mediated anterograde and retrograde trafficking modulates phagosome maturation and contributes to the restriction of the bacterial growth, leading to the killing of mycobacteria.

Figure S9-IFN-γ released from splenic or lymph node lymphocytes in *Sort1*$^{+/+}$ (WT) and *Sort1*$^{-/-}$ (KO) mice.

A- IFN-γ levels from splenic lymphocytes stimulated with the indicated concentration of anti-CD3 in presence (left panel) and absence (right panel) of IL-12. **B-** Levels of IFN-γ in splenic and lymph node lymphocytes obtained from *Sort1*$^{+/+}$ and *Sort1*$^{-/-}$ mice. Data show a representative experiment performed in triplicate.

Movie S1- RAW 267.4 cell transfected with Sort/WT-EYFP and infected with *M. bovis* BCG-DsRed. Scale bar: 10 μm. Images were acquired during 17 minutes and each image was acquired every 20 seconds. This video corresponds to Fig. 1G.

Supplementary Material and Methods

Indirect immunofluorescence

RAW 267.4 cells, BMM or THP-1 cells (depending on the experiment) were fixed with 3 % paraformaldehyde solution in PBS for 15 minutes at room temperature, washed with PBS and quenched with glycine 50 mM in PBS for 20 minutes, pH 7.4 (Sigma). Then, cells were permeabilized with 1 % saponin in PBS containing

1 % BSA (blocking buffer). After blocking, cells were incubated with the indicated primary antibodies for 1 h or overnight. Cells were washed three times in blocking buffer and subsequently incubated with the indicated fluorophore-conjugated secondary antibody for 1 h. DNA staining was performed using Hoechst 33258 in PBS during 10 minutes (1 µg/mL, Sigma, Germany). Coverslips were mounted on slides with DAKO mounting medium (Dako Cytomation, Denmark).

SDS-PAGE and Western blot analysis

To detect level of expression of the sortilin wild type and mutants, RAW 267.4 cells were transfected with the different sortilin constructs as indicated in material and methods. Cells were washed, scraped, resuspended in sample buffer containing 2-mercaptoethanol 1 % and sonicated for 10 min at 4 °C. The samples were frozen until use. For western blots analysis, the protein lysates were subjected to electrophoresis in 10 % SDS-PAGE gel, transferred to a nitrocellulose membrane and blocked with PBS supplemented with 0.1 % v/v of Tween-20 and 5 % of milk. The nitrocellulose membrane was incubated for 2 h with a rabbit anti-GFP (Abcam, USA) (1:200), washed and incubated with a secondary HRP-conjugated goat anti-rabbit antibody (Jackson ImmunoResearch Laboratories, Inc) at a 1:10000 dilution. Equal proteins loading were confirmed using mouse anti-tubulin antibody (Hybridoma Bank, Iowa, USA) (1:2000) and a secondary HRP-conjugated goat anti-mouse antibody (Jackson ImmunoResearch Laboratories, Inc) at a 1:10 000 dilution was used to detect the bands. The bands were visualized using the ECL reagent (GE Healthcare, Buckinghamshire, UK), and analyzed using t “Gels” function in the tools of Fiji software.

IFN-γ ELISA

Lymphocytes were obtained as described before [1]. IFN-γ was measured by ELISA using cytokine capture and biotinylated detection antibodies (R&D Systems). The concentration of IFN-γ was determined from a serial-fold diluted standard curve with OD read at 450 nm in an ELISA reader.

Supplementary References

1. Okoye IS, Coomes SM, Pelly VS, Czieso S, Papayannopoulos V, Tolmachova T, Seabra MC, Wilson MS. MicroRNA-containing T-regulatory-cell-derived exosomes suppress pathogenic T helper 1 cells. *Immunity*. **41(1)** 89-103, doi: 10.1016/j.immuni.2014.05.019. (2014).

Figure S1

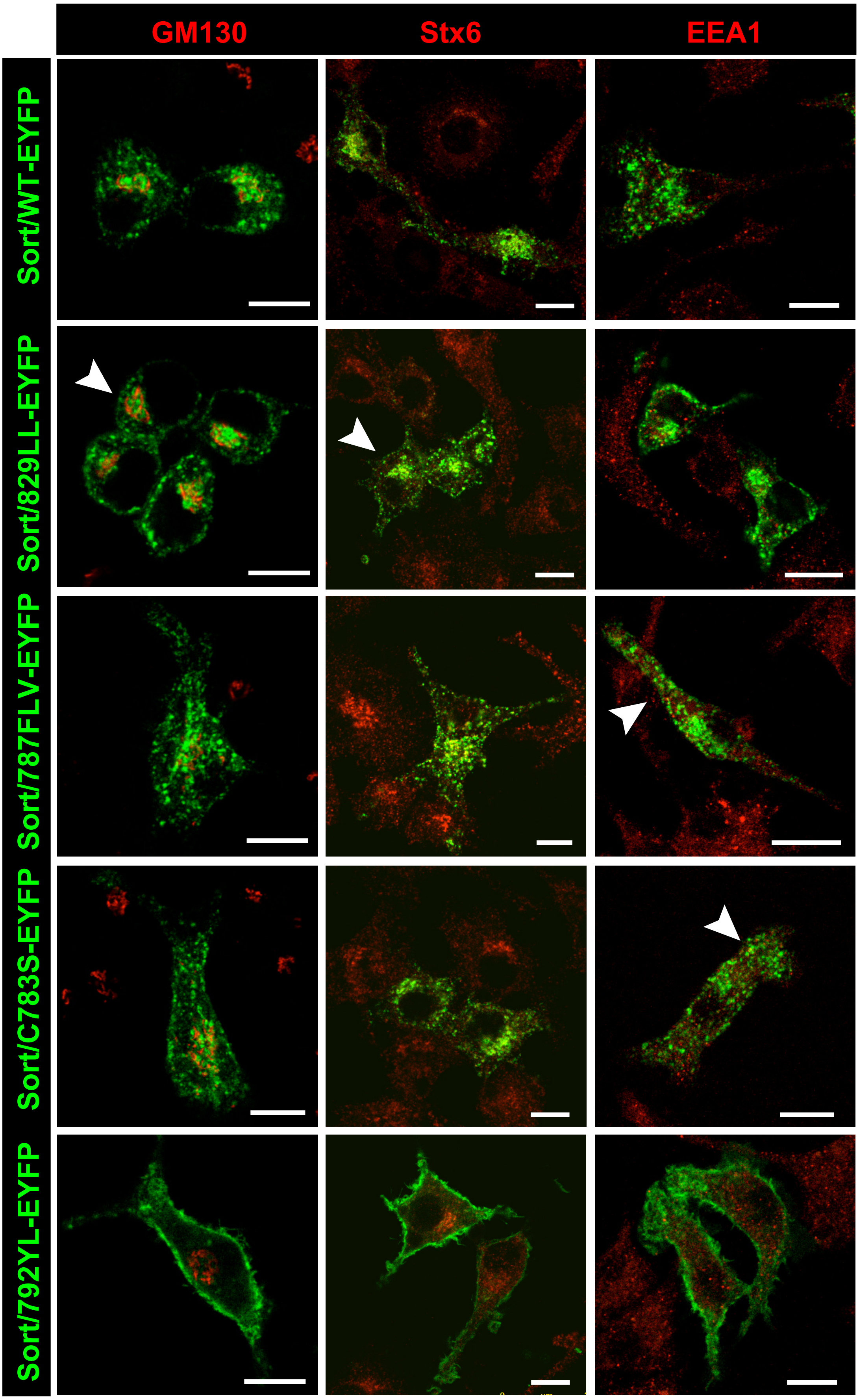
GM130
Stx6
EEA1
Sort/WT-EYFP
Sort/829LL-EYFP
Sort/787FLV-EYFP
Sort/C783S-EYFP
Sort/792YL-EYFP

Figure S2

A

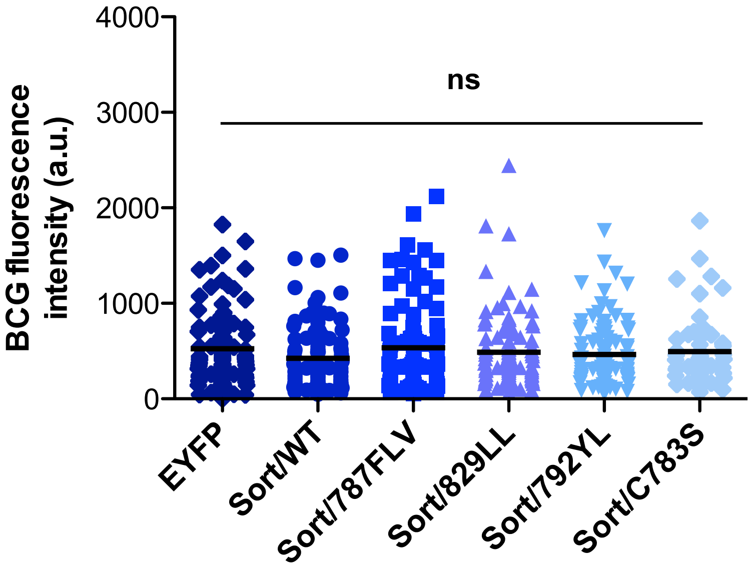

4000
3000
2000
1000
0
BCG fluorescence intensity (a.u.)
ns
EYFP
Sort/WT
Sort/787FLV
Sort/829LL
Sort/792YL
Sort/C783S

Figure S3

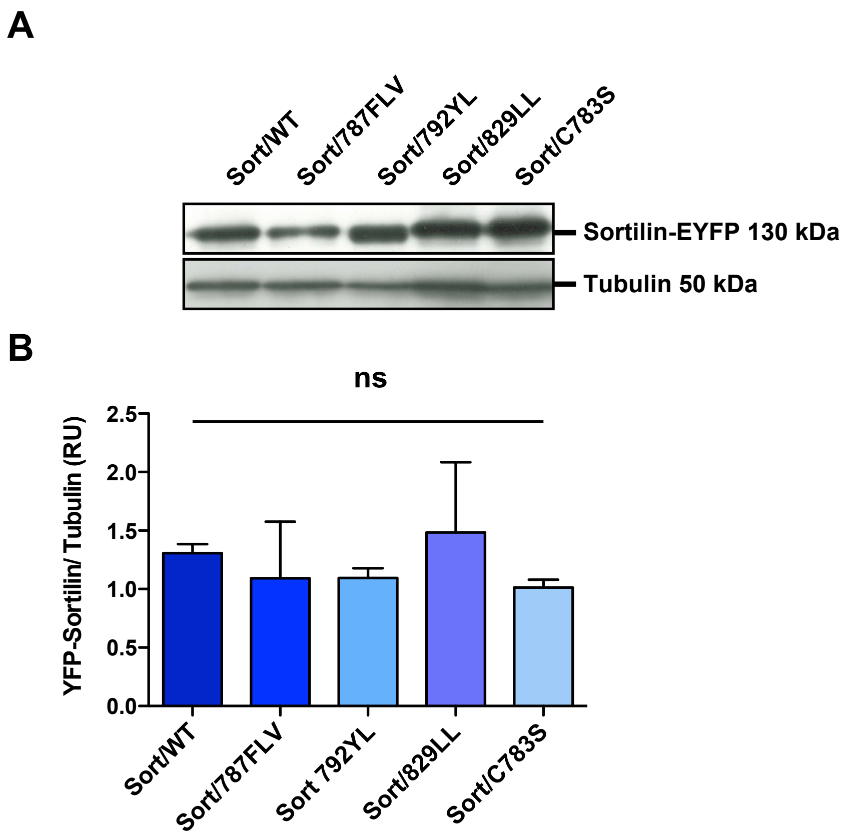
A
Sort/WT
Sort/787FLV
Sort/792YL
Sort/829LL
Sort/C783S
Sortilin-EYFP 130 kDa
Tubulin 50 kDa
B
ns
YFP-Sortilin/ Tubulin (RU)
2.5
2.0
1.5
1.0
0.5
0.0
Sort/WT
Sort/787FLV
Sort 792YL
Sort/829LL
Sort/C783S

Figure S4

A

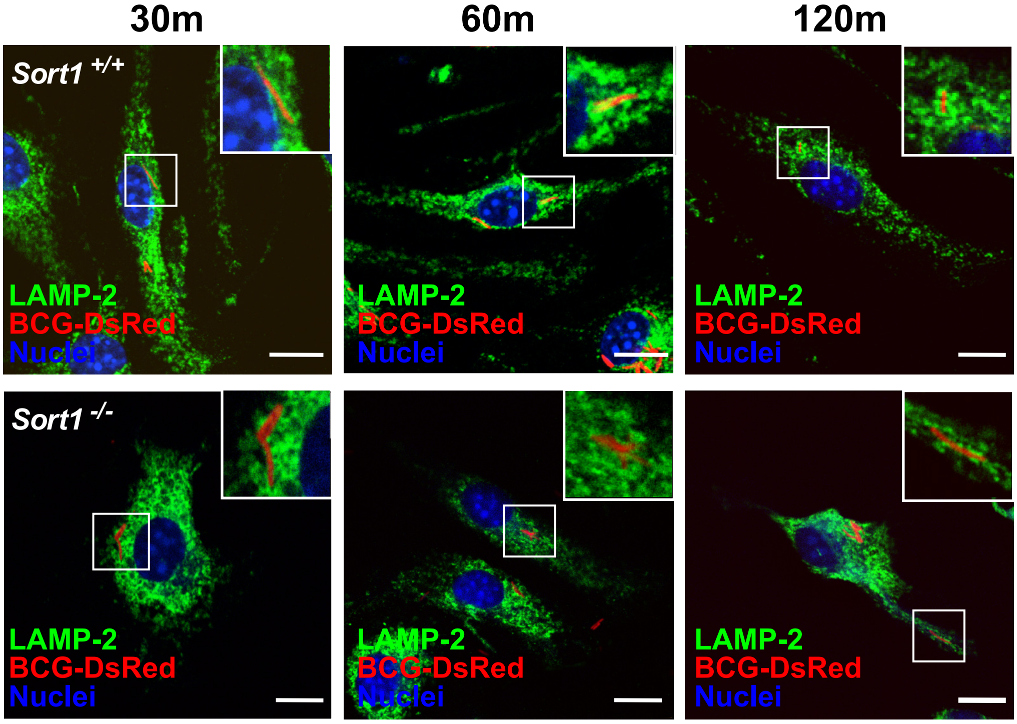
30m
60m
120m
Sort1 +/+
Sort1 -/-
LAMP-2
BCG-DsRed
Nuclei

B

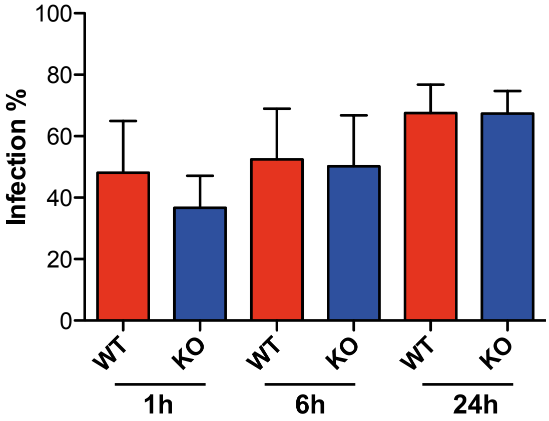
Infection %
100
80
60
40
20
0
WT
KO
1h
6h
24h

Figure S5

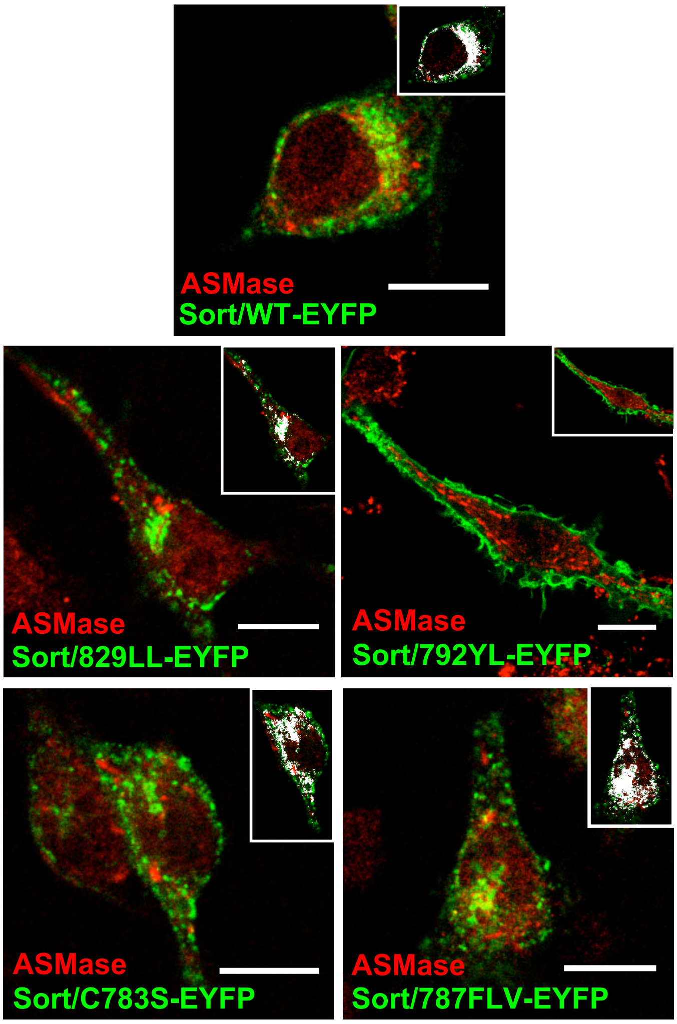
ASMase
Sort/WT-EYFP
ASMase
Sort/829LL-EYFP
ASMase
Sort/792YL-EYFP
ASMase
Sort/C783S-EYFP
ASMase
Sort/787FLV-EYFP

Figure S6

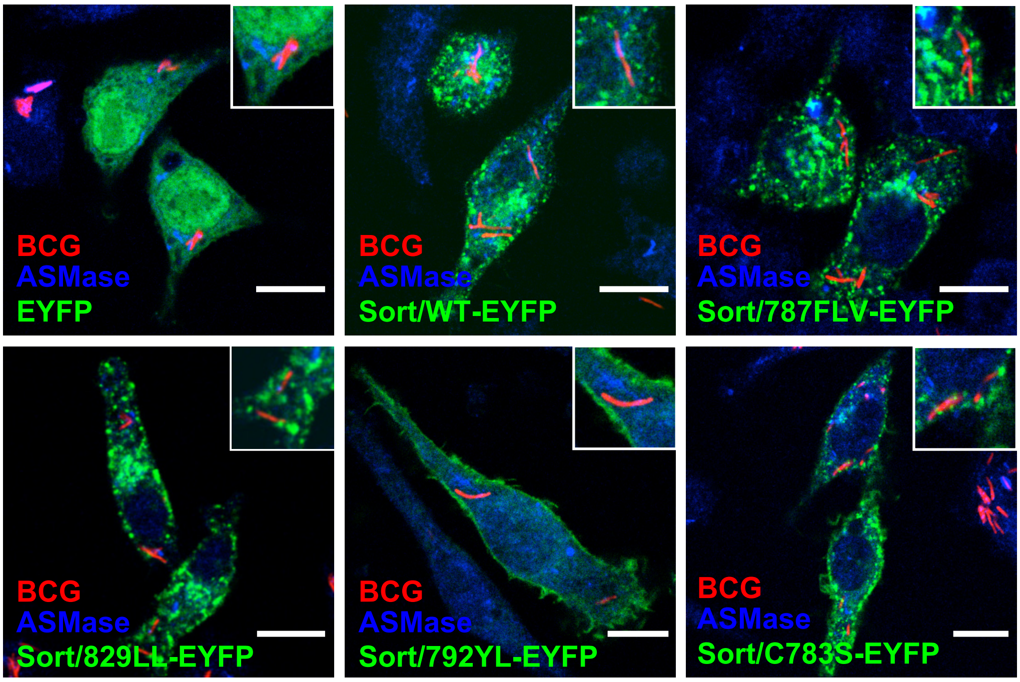
BCG
ASMase
EYFP
BCG
ASMase
Sort/WT-EYFP
BCG
ASMase
Sort/787FLV-EYFP
BCG
ASMase
Sort/829LL-EYFP
BCG
ASMase
Sort/792YL-EYFP
BCG
ASMase
Sort/C783S-EYFP

Figure S7

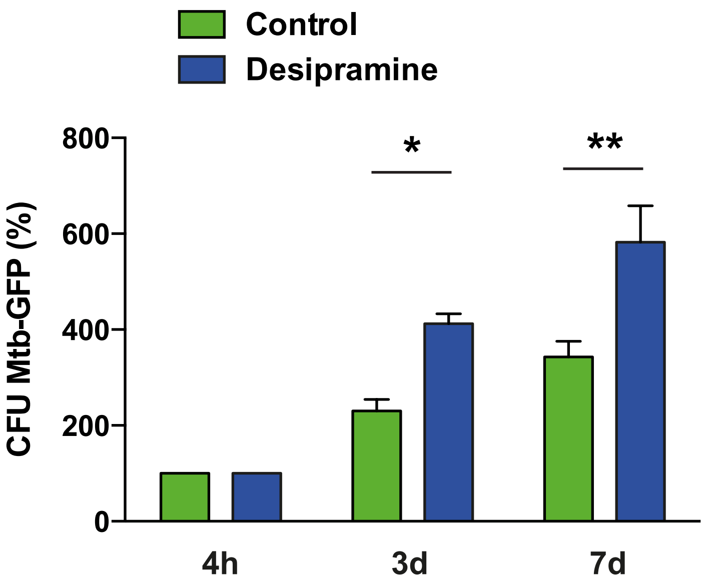

Figure S8

Proposed model of the function of sortilin in mycobacterial phagosomes

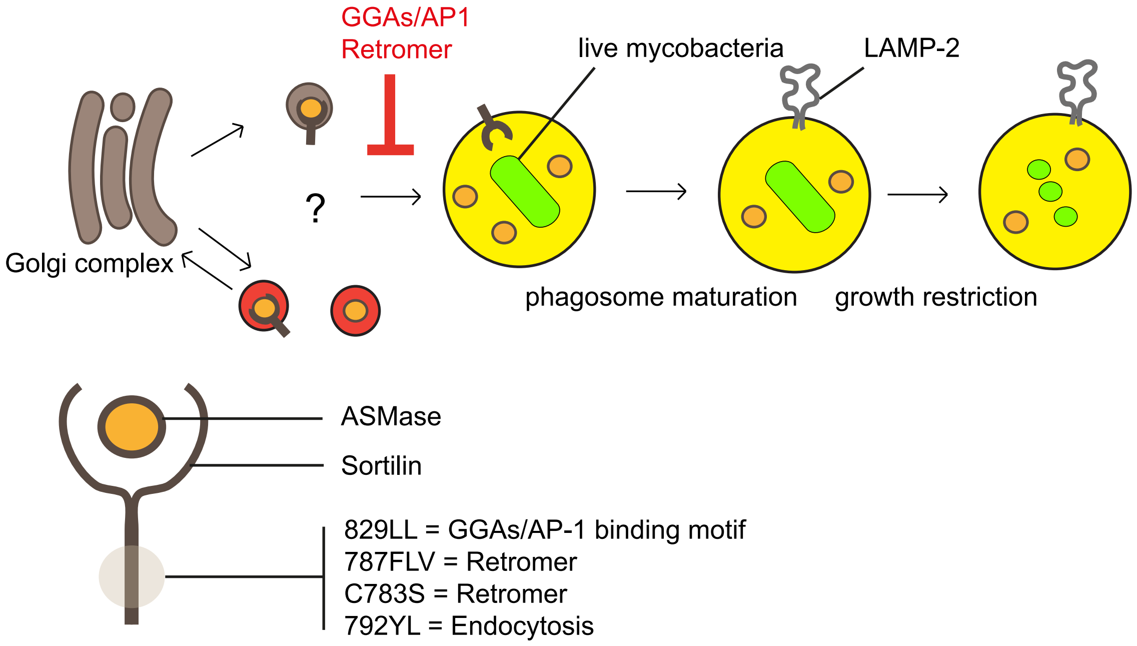

Figure S9

A

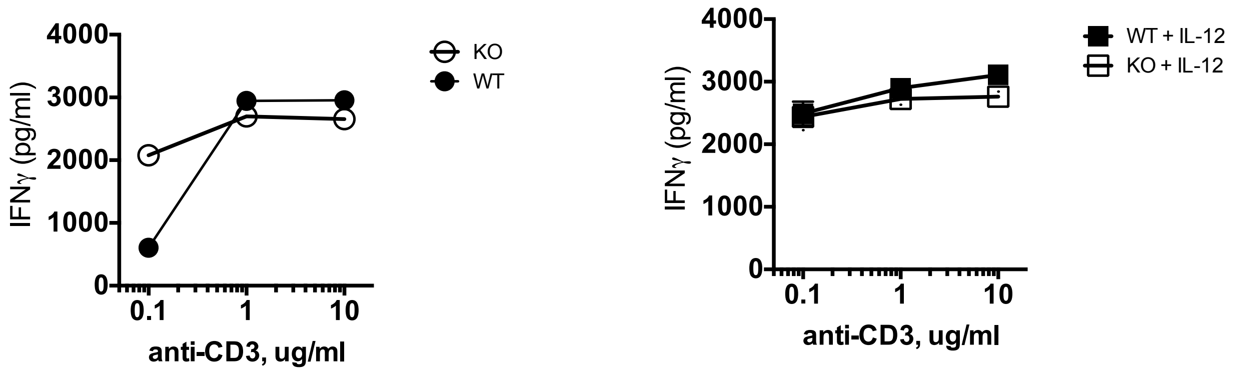

IFNγ (pg/ml)
4000
3000
2000
1000
0
0.1
1
10
anti-CD3, ug/ml
KO
WT
WT + IL-12
KO + IL-12

B

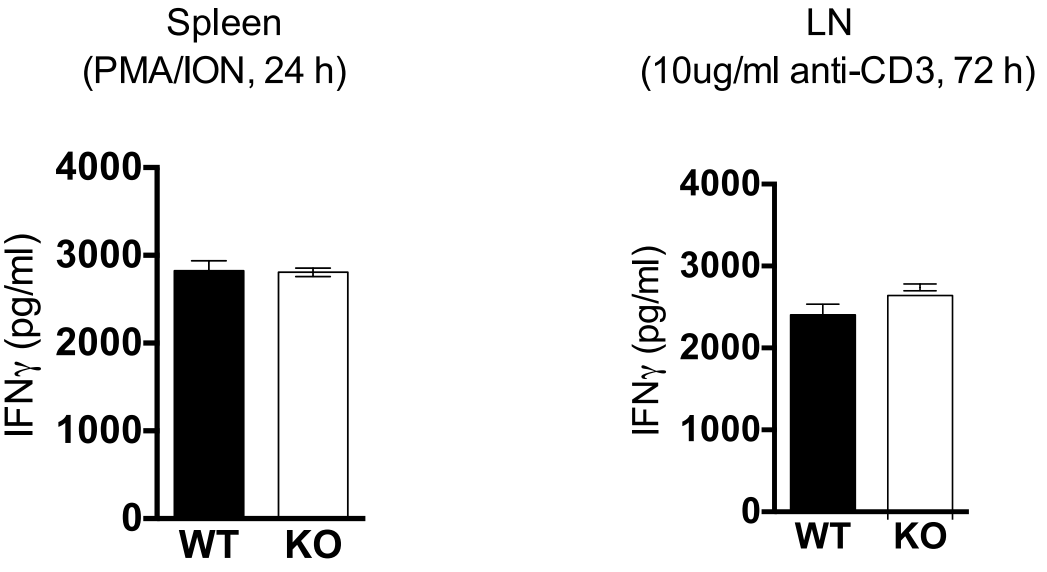

Spleen
(PMA/ION, 24 h)
LN
(10ug/ml anti-CD3, 72 h)
IFNγ (pg/ml)
4000
3000
2000
1000
0
WT
KO
